# Contrast enhancement in the mammillary bodies: An easily missed sign of Korsakoff syndrome

**DOI:** 10.1590/1980-57642015DN94000422

**Published:** 2015

**Authors:** Fábio H.G. Porto, Jessica Harder, Gislaine C.L. Machado-Porto

**Affiliations:** 1 Centro de Referência em Distúrbios Cognitivos (CEREDIC) do Hospital das Clínicas da Faculdade de Medicina da Universidade de São Paulo (HC/FMUSP), São Paulo, SP, Brazil.; 2 Brigham and Women's Hospital, Boston, MA, USA.; 3 Department of Radiology, AC Camargo Cancer Center, São Paulo SP, Brazil.

**Keywords:** Korsakoff syndrome, MRI, mammilary bodies, diagnosis, síndrome de Korsakoff, ressonância magnética, corpos mamilares, diagnóstico

A 75-year-old man was evaluated for cognitive dysfunction noticed by his daughter during a telephone call. The patient had a past history of hypertension, dyslipidemia, depression, blindness in the right eye due to trauma, alcohol abuse and smoking. He lived alone and was totally independent. There was no previous cognitive dysfunction reported. According to reports by his relatives, the patient had slurred speech and seemed confused and unsteady the day following the telephone call. A few days later he was hospitalized after a fall, with a mild traumatic brain injury. During the hospital stay he was diagnosed with pneumonia and received treatment with antibiotics. After discharge, he was cognitively changed. He had rapid forgetfulness, and was unable to learn new information. He also had some difficulties with past memories, frequently reporting information that was not true. His habitual behavior had changed. He was apathetic but when his relatives tried to stimulate him, he became irritated and aggressive. He frequently thought that he was still working and tried to go to work, despite having retired long ago. When his family attempted to stop him, he became agitated. The patient was evaluated about two months after the onset of his illness.

Elementary neurologic examination disclosed a slightly unstable wide-based gait, but was otherwise unremarkable. There were no alterations in eye motility, appendicular ataxia or peripheral neuropathy. Results of cognitive evaluation showed a score on the Mini-Mental State Examination of 14 with problems recalling the three words. Even after several trials to learn the words, it was impossible for him to remember them after a few minutes. A brief cognitive battery also disclosed severe anterograde amnesia. Score on the functional activities questionnaire was 30 (maximal score for functional disability). Laboratory investigation disclosed only folic acid and vitamin D deficiencies. Level of thiamine (B1) was normal. MRI showed hyperintensities in the medial thalamus and periaqueductal gray matter, and a contrast enhancement in both mammillary bodies (Figure). The diagnosis of Korsakoff syndrome (KS) was reached and supplementation with high dose thiamine was started. In spite of the supplementation of thiamine, the symptoms did not improve and the introduction of antipsychotics was needed to control his behavior. A trial of cholinesterase inhibitor was also attempted.

**Figure 1:**
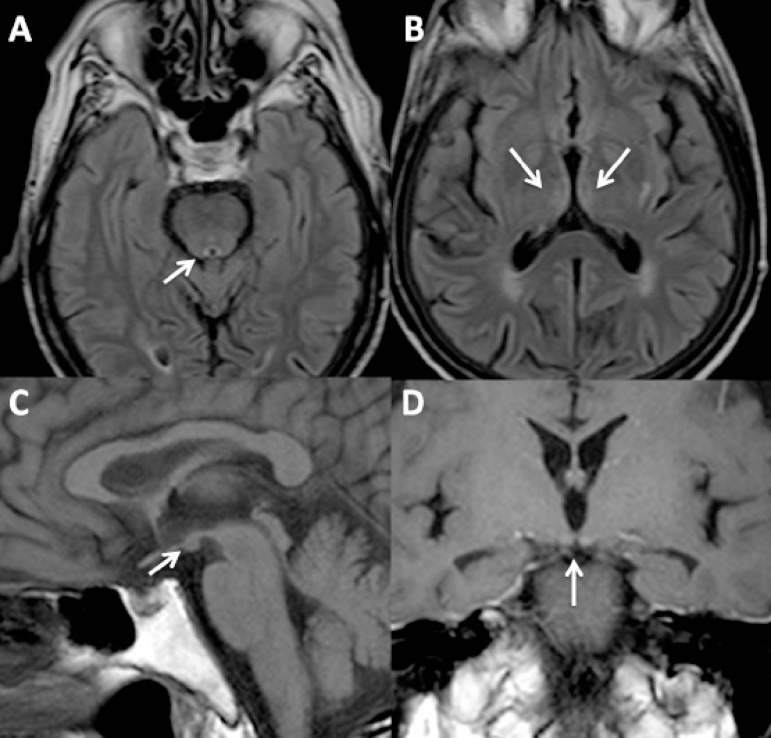
[A and B] FLAIR images depicting hyperintensities in the periaqueductal region [A] and medial thalamus [B]. Pre- [C] and Post- [D] contrast T1-weighted images showing contrast enhancement of the mammillary bodies.

KS is a neurological disorder characterized by severe memory and learning deficits, associated with thiamine deficiency, usually (but not always) associated with alcohol misuse and malnutrition.^1,2^ The condition often follows undiagnosed or inadequately treated Wernicke encephalopathy (WE). WE is an acute neuropsychiatric syndrome characterized by mental status changes, disturbances in eye motility (nystagmus and ophthalmoplegia), gait ataxia and unsteadiness of stance. It is also associated with thiamine deficiency.

KS is associated with lesions in specific brain areas that are vulnerable to thiamine depletion because of their high thiamine utilization and turnover.^3^ The anterior and mediodorsal nuclei of the thalamus, mammillothalamic tract and mammillary bodies number among these regions. They are important regions of the anterograde memory circuit and explain why memory impairment is the main feature of KS.

Because there is no specific routine laboratory test for the diagnosis of KS and WE, neuroimaging is of great value in suspected cases. MRI may depict elevated T2-signal bilaterally in thalamic nuclei, hypothalamus, mammillary bodies, the periaqueductal region, the floor of the fourth ventricle and midline cerebellum.^4^ Contrast enhancement can be seen, showing disruption of the blood-brain barrier. Sometimes, contrast-enhancement in the mammillary bodies may be the only imaging sign of KS. This can be easily missed by neurologists unfamiliar with the neuroimaging features of the disease. In our case report, neuroimaging features were very helpful, because the patient was first seen after two months and the encephalopathic state was not seen. Also, serum level of thiamine was normal, reflecting the lack of sensitivity of its concentration. The contrast enhancement of the mammillary bodies probably resolves sometime after the acute WE, and atrophy may appear later.
